# BE-BOIN: A Dose Optimization Design Accommodating Backfill and Late-Onset Toxicity

**DOI:** 10.1007/s43441-026-00994-0

**Published:** 2026-08-10

**Authors:** Kai Chen, Yixuan Zhao, Kentaro Takeda, Ying Yuan

**Affiliations:** 1 Eli Lilly and Company, Indianapolis, IN USA; 2 Department of Biostatistics and Data Science, The University of Texas Health Science Center, Houston, TX USA; 3 Astellas Pharma Global Development Inc., Northbrook, IL USA; 4 Department of Biostatistics, The University of Texas MD Anderson Cancer Center, Houston, TX USA

**Keywords:** Project optimus, Dose finding, Backfill, Late-onset toxicity, BOIN design

## Abstract

The US Food and Drug Administration (FDA) launched Project Optimus and issued guidance to reform dose-finding and selection trials, shifting the paradigm from identifying the maximum tolerated dose (MTD) to determining the optimal biological dose (OBD), which optimizes the risk and benefit of treatments. The FDA’s guidance emphasizes the importance of collecting sufficient toxicity and efficacy data across multiple doses and considering late-onset cumulative toxicity that often results in tolerability issues. To address these challenges, we propose the BE-BOIN (Backfill time-to-Event Bayesian Optimal INterval) design, which allows backfilling patients into safe and effective doses during dose escalation and accommodates late-onset toxicities. BE-BOIN enables the collection of additional safety and efficacy data to enhance the accuracy and reliability of OBD selection and supports real-time dose decisions for new patients. Our simulation studies show that BE-BOIN accurately identifies the MTD and OBD while significantly reducing trial duration.

**Supplementary Information:**

The online version contains supplementary material available at https://doi.org/10.1007/s43441-026-00994-0.

## Introduction

The FDA launched Project Optimus and issued guidance [1] to shift the dose finding and selection paradigm from finding the maximum tolerated dose (MTD) to identifying the optimal biological dose (OBD), which aims to balance efficacy and safety to optimize the risk-benefit profile of investigational drugs. Accordingly, the focus of dose-finding trials should shift from the conventional approach of escalating the dose to the highest tolerable level to comparing multiple dose levels in terms of risk and benefit, as emphasized in the FDA’s guidance. Achieving this goal requires collecting a reasonable amount of data across multiple doses. Yuan et al. [2] and Huang & Yuan [3] provide a comprehensive review of these methods, including efficacy-integrated designs, two-stage designs, and seamless phase 2-3 designs [4]. In particular, the FDA guidance highlights backfilling as a useful strategy to collecting more data across multiple doses, stating: “It may be useful to evaluate additional dose-level cohorts or add more patients to existing dose-level cohorts (i.e., backfill cohorts) in the dose-finding trial for dosages being considered for further development. This would provide additional clinical data to allow for further assessment of safety and activity prior to initiating a trial to compare multiple dosages".

A number of methods have been proposed to incorporate backfilling into phase I trials, which involve concurrently assigning patients to doses previously deemed safe and showing preliminary efficacy during escalation. Dehbi et al. [5] proposed randomizing patients to backfill doses based on hypothesis testing. Barnett et al. [6] studied the effect of backfill on the estimation of the MTD and the trial duration based on Bayesian logistic regression model. Chen et al. [7] proposed a Bayesian design that uses patient-reported outcomes to determine doses for backfill. Pin et al. [8] considered using response-adaptive randomization to allocate patients among backfill doses. Zhao et al. [9] proposed the BF-BOIN design, which seamlessly integrates backfill into the BOIN framework with formal statistical criteria for opening and closing backfill cohorts and resolving potential data conflicts between dose escalation and backfill. Compared to other backfill designs, such as Dehbi et al. [5] and Barnett et al. [6], which require complex model fitting and estimation, a key advantage of BF-BOIN is its ability to preserve the hallmark simplicity and robust performance of BOIN. The BF-BOIN design has already been implemented in a number of clinical trials (see, for example, ClinicalTrials.gov identifiers NCT05902988, NCT06908434, and NCT06463340). Subsequently, Zhao et al. [10] proposed the BARD design that enables the pooling of dose escalation and backfill patients with subsequent randomized patients to efficiently inform the selection of the OBD.

Another important issue that motivated Project Optimus and is emphasized in the FDA’s guidance is the late-onset toxicity and tolerability issue. The toxicity of targeted therapies is often late-onset [11–13]. For example, Postel-Vinay et al. [11] reported that in 36 clinical trials of molecularly targeted agents, 57% of grade 3–4 toxicities occurred after the first treatment cycle. Additionally, as noted by Shah et al. [14] and Fourie Zirkelbach et al. [15], acute DLT is seldom observed in many novel targeted agents. Instead, as these agents are often administered over multiple cycles, they frequently result in cumulative late-onset toxicities, causing tolerability issues that lead to dose interruption, reduction, and discontinuation. To capture these late-onset toxicities, it is imperative to use a longer toxicity observation window. This poses tremendous logistical and operational challenges for sequential dose-finding designs, which typically require fully observed data before making dosing decisions for the next cohort of patients.

Several designs have been proposed to address late-onset toxicity, including model-based designs such as TITE-CRM [16], an extension of the continual reassessment method (CRM) [17], DA-CRM [18], and EM-CRM [19], as well as model-assisted designs like TITE-BOIN [20] and TITE-keyboard [21]. These designs account for patients who are still within the toxicity assessment window (referred to as "pending patients") by leveraging their follow-up data to enable real-time dose decisions for new cohorts. For instance, TITE-CRM incorporates pending patients by weighting their likelihood based on the fraction of their toxicity window that has been completed. The TITE-BOIN design imputes pending toxicity data for pending patients using their follow-up data and combines these imputed data with observed data to make real-time decisions. TITE-BOIN has been successfully applied in various trials, including those for targeted therapies (ClinicalTrials.gov ID: NCT04388852), immunotherapies (NCT03956680), radiotherapies (NCT06617169), and chemotherapies (NCT05620654).

In this article, we propose BE-BOIN (Backfill TITE-BOIN), an extension of BF-BOIN that accommodates late-onset toxicity using the TITE-BOIN approach. We provide the rules for opening and closing backfill, as well as strategies for addressing potential conflicts when backfill doses exhibit higher toxicity rates than the current dose-escalation dose. A unique challenge in this setting is incorporating pending toxicity outcomes from both dose-escalation and backfill patients into decision making. To address this, we adopt the imputation approach from TITE-BOIN to handle pending data effectively. Simulation studies demonstrate that BE-BOIN exhibits desirable operating characteristics, including shorter trial durations compared to BF-BOIN, the ability to treat more patients than TITE-BOIN, and often improved accuracy in identifying the MTD and OBD. As a byproduct, our results also demonstrate that the BF-BOIN approach of ignoring pending backfilling patients has a negligible impact on the operating characteristics of the design, addressing a common question raised about BF-BOIN.

The remainder of this article is organized as follows. Section 2 introduces the time-to-event model and the BE-BOIN design. Section 3 presents the simulation study, and the article concludes with a discussion in section 4.

## Methods

### Handling Pending Data

A key challenge in trials involving late-onset toxicity and backfill is managing pending data, which hinders real-time dose-decision-making for the next cohort of patients. Pending data arise not only from patients in dose escalation but also from those in backfill. In the following sections, we first discuss methods for handling pending data, followed by the design decision rules.

Assume there are *J* dose levels, and let $p_j$ denote the dose-limiting toxicity (DLT) rate of dose *j*, where *j = 1, 2, … , J.* Suppose at an interim decision time, $n_j$ patients have received dose *j*. Let *(0,τ )* denote the DLT assessment window, and let $x_i$ denote the DLT indicator for patient *i* ($i=1, \cdots , n_j$), with $x_i=1$ indicating DLT and $x_i=0$ indicating no DLT within *(0, τ )*. The DLT window *τ* is typically prespecified and should be sufficiently wide to capture all DLTs relevant to dose-escalation decisions. In oncology, the first treatment cycle (e.g., 21 or 28 days) is commonly used as the DLT assessment window. However, for therapies with potentially late-onset toxicities (e.g., radiotherapy or certain immunotherapies), *τ* may be extended to two or more cycles to ensure that all relevant DLTs are adequately observed. The issue of modifying the DLT window during a trial will be briefly discussed in the Discussion section.

At the interim time, let $O_j$ represent the group of patients who have completed the DLT assessment (i.e., for whom $x_i$ is known), and $M_j$ represent the group of patients whose DLT assessments are still pending (i.e., $x_i$ is unknown). We estimate $p_j$ as:

$$\hat{p}_j = \frac{\sum _{i \in O_j} x_i + \sum _{i \in M_j} x_i}{n_j}.$$

The challenge in calculating $p_j$ is that $x_i \in M_j$ is unobserved.

Let $u_i$ denote the time to DLT, and $t_i$ denote the follow-up time for patient *i* at the interim decision time. Following the TITE-BOIN [20], and assuming $u_i$ follows a uniform distribution over the DLT assessment window *(0,τ )* for patients with $x_i=1$, the unobserved $x_i \in M_j$, with a follow-up time $t_i \le \tau$, can be imputed using its expectation:

$$\begin{aligned} \hat{x}_i&= E(x_i | u_i>t_i) = \Pr (x_i =1|u_i>t_i ) \\&= \frac{\Pr (u_i>t_i |x_i=1)\Pr (x_i=1)}{\Pr (u_i>t_i |x_i=1)\Pr (x_i=1) + \Pr (u_i>t_i |x_i=0)\Pr (x_i=0)} \\&=\dfrac{(1-\dfrac{t_i}{\tau })p_j}{(1-\dfrac{t_i}{\tau })p_j + (1-p_j)} \approx \dfrac{(1-\dfrac{t_i}{\tau })p_j}{1-p_j}. \end{aligned}$$

The rationale for the approximation is that $p_j$ and consequently (1-${t_i}/{\tau })p_j$ are often small relative to $1-p_j$. After the imputation, $\hat{p}_{j}$ is given as:

1 $$\begin{aligned} \hat{p}_j = \frac{\tilde{y}_j + \dfrac{p_j}{1-p_j}(m_j - T_j)}{n_j}, \end{aligned}$$

where $\tilde{y}_j = \sum _{i \in O_j}x_i$ is the number of observed DLTs, $m_j$ is the total number of pending patients, and $T_j=\sum _{i \in M_j} {t_i}/{\tau }$ is the standardized total follow-up times for the $m_j$ pending patients at dose *j*. To calculate $\hat{p}_{j}$, the unknown $p_j$ can be replaced by its Bayesian posterior mean estimate, $\tilde{p}_j=(\alpha + \tilde{y}_j)/(\alpha + \beta + n_j -m_j)$, obtained from a beta-binomial model with the prior $p_j \thicksim \text {Beta}(0.5\phi ,1-0.5\phi )$. This vague prior corresponds to an effective prior sample size of 1, with the prior mean equal to *0.5ϕ*, where *ϕ* is the target DLT rate.

Following the same approach, we can also calculate the pooled toxicity rate $\hat{q}_{(b,k)}$ across doses from *b* to a higher *k*, where $1\le b < k \le J$,

2 $$\begin{aligned} \hat{q}_{(b,k)} = \frac{\sum _{j = b}^k \tilde{y}_j + \sum _{j = b}^k\dfrac{\tilde{p}_j}{1-\tilde{p}_j}(m_j -T_j) }{\sum _{j = b}^k n_j}. \end{aligned}$$

$\hat{q}_{(b,k)}$ will be utilized to select eligible doses for backfilling and to reconcile data conflicts arising from violations of toxicity monotonicity, as discussed in the next section.

### Trial Design

**Fig. 1:**
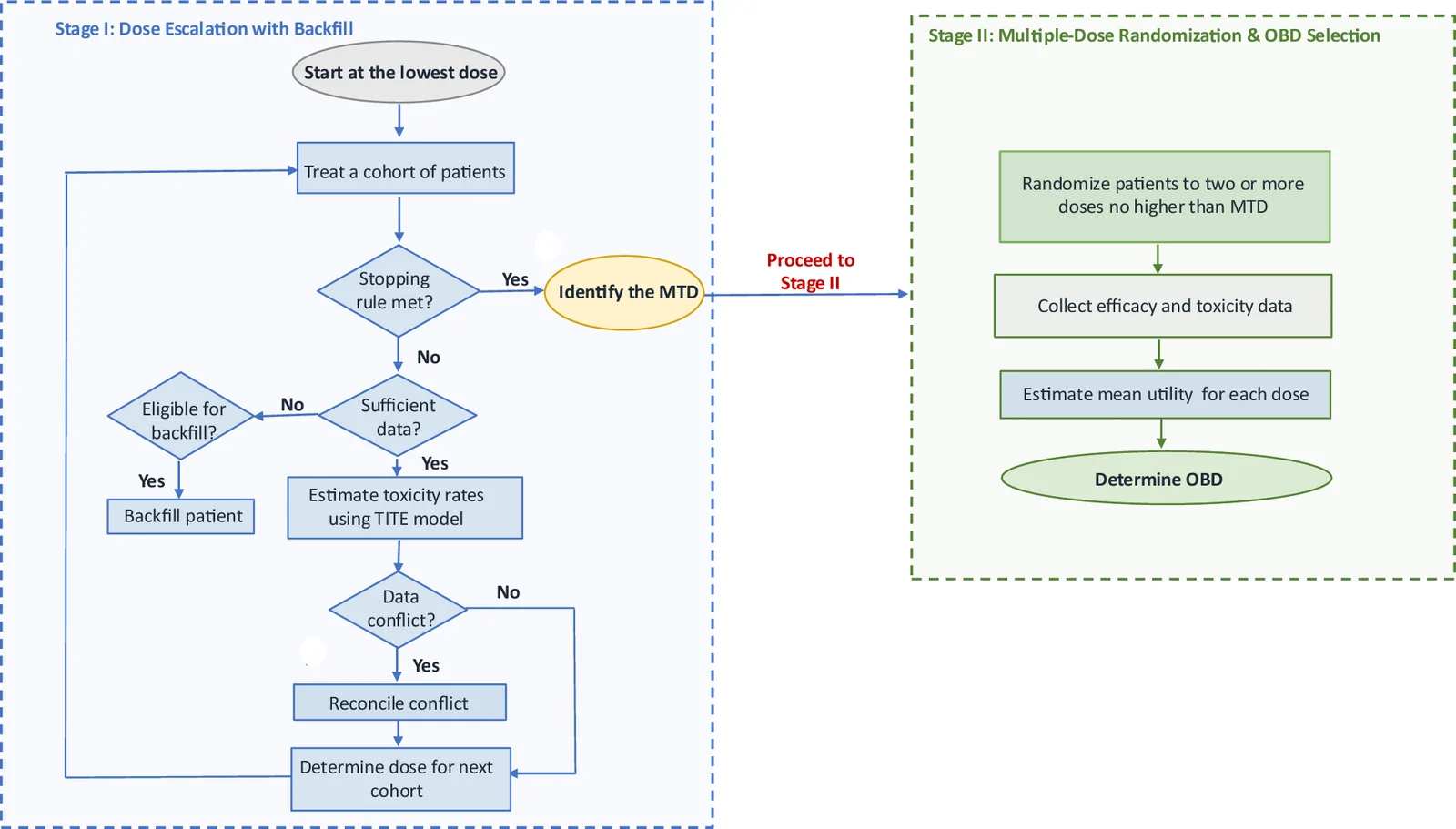
Flowchart of the BE-BOIN design. The flowchart illustrates the prioritization of patient accrual for dose escalation; however, this prioritization is not mandatory. Backfilling can be initiated concurrently when “sufficient data" are available to inform dose-escalation decisions, i.e., backfilling is not strictly contingent upon a pause in the dose-escalation phase

The BE-BOIN design consists of two interconnected components: dose escalation (DE) and backfill. In the DE component, BOIN design rules are applied to explore the dose space and identify the MTD by the end of the trial. The backfill component is opened when a dose is deemed safe and demonstrates preliminary activity based on data from DE. Once opened, additional patients are concurrently treated at one of these doses while DE continues. At both interim and final analyses, cumulative data from DE and BF are combined to guide decision-making. Figure 1 presents the flowchart of the BE-BOIN design. In the following sections, we describe DE, followed by backfill.

#### Dose Escalation

In DE, patients are treated in cohorts. The most commonly used cohort size is three, but it can be adjusted based on the characteristics of the trial. Suppose at a dose decision time for the next cohort, and current cohort is treated at dose *c* in DE. Let $\lambda _e$ and $\lambda _d$ denote escalation and de-escalation boundaries of the BOIN design [22, 23]. BE-BOIN determines dose escalation or de-escalation decisions as follows:

1. Treat the first cohort of patients at the lowest dose or at pre-specified doses.
2. To assign a dose to the next cohort of patients, calculate $\hat{p}_c$ using equation (1),
  - If $\hat{p}_c \le \lambda _e$, escalate to dose *c+1*;
  - If $\hat{p}_c> \lambda _d$, de-escalate to dose *c-1*;
  - Otherwise: retain the current dose *c*.
3. Repeat step 2 until the maximum sample size *N* is reached.

A rule of thumb for determining the maximum sample size *N* in step 3 is to set it as *6× J* [24]. For instance, in a phase I trial with five dose levels, *N* would be set to 30. This value can be further calibrated to ensure desirable operating characteristics.

As a tradeoff for the ability to make decisions in the presence of pending patients, the described DE approach is associated with greater uncertainty compared to fully staggered designs, as patients with pending DLT outcomes might later exhibit a DLT after a decision has already been made [25]. This uncertainty increases with the number of pending patients at the time of dose decision-making. To mitigate this concern and ensure decisions are made based on adequate data and follow-up time, following Chen et al. [26], we implement two accrual suspension rules:

**Accrual Suspension Rule 1**: Suspend accrual to wait for more DLT data if fewer than 51% of patients at the current dose have their DLT outcome observed, unless the observed DLT rate has already exceeded $\lambda _d$.

**Accrual Suspension Rule 2**: Suspend accrual to allow for longer follow-up of pending patients if the data recommends dose escalation and the shortest follow-up time for pending patients at the current dose is less than 25% of the DLT assessment window.

Rule 1 requires that a dose-escalation or stay decision be made only when more than half of the patients at the current dose have completed the safety assessment window. This ensures that decisions are based on sufficient information and remain consistent with common practices in phase I trials. In particular, when six patients are treated at the current dose, setting the threshold at $\le 50\%$ would allow a dose escalation if three patients have completed their DLT assessment with no observed DLTs, even though the remaining three patients are still pending. If two or three of the pending patients later experience DLTs after escalation, patient safety could be compromised. Using a threshold of 51% mitigates this risk. Rule 2 further safeguards against premature escalation by prohibiting dose-escalation decisions when pending patients have limited or no follow-up data. In practice, investigators and regulatory authorities often prefer that pending patients be followed for a minimum period before escalation to ensure adequate safety information.

Chen et al. [26] conducted a comprehensive simulation study and recommended thresholds of 51% for Rule 1 and 25% for Rule 2. These values strike an appropriate balance between minimizing trial duration and maintaining patient safety. However, they are adjustable and can be calibrated to meet the specific objectives of each trial.

One prominent advantage of the TITE-BOIN framework is that the dose escalation/de-escalation rules described above can be pre-tabulated prior to trial initiation (see Supplementary Material S1 for an example). Consequently, dose escalation and de-escalation decisions can be easily made by referring to the decision table, greatly simplifying its implementation. This contrasts with other methods, such as TITE-CRM, which require repeated model fitting and estimation. A free online tool for generating the decision table, available as part of the BOIN Suite, is available at https://www.trialdesign.org.

#### Backfill Patients

In the DE component, if suspension rules are triggered due to a high number of pending patients or insufficient follow-up time, the accrual of new patients to the DE should be paused. In such instances, newly enrolled patients may be backfilled to lower, safe dose levels that have already demonstrated reasonable efficacy. Generally, backfilling is not contingent upon a pause in DE; it can proceed concurrently with the DE phase to gather additional data for a more precise assessment of the dose-toxicity and dose-efficacy relationships. However, when accrual to the DE is paused, backfilling provides the added benefit of ensuring accrual continuity and maintaining patient access to the trial, which can potentially shorten the overall trial duration. This feature is particularly advantageous in settings involving late-onset toxicity or an extended DLT evaluation window, where newly enrolled patients might otherwise face significant delays before being assigned to the DE.

To backfill patients, only doses showing both efficacy and safety are eligible for backfilling. Following Zhao et al. [9], given the current DE dose *c*, a lower dose *b* *(b<c)* is considered eligible for backfilling if it meets the following three criteria:

- Safety: $\hat{p}_{b} \le \lambda _d$, or $\hat{p}_{b}> \lambda _d$ but the pooled estimate $\hat{q}_{(b, b+1)} \le \lambda _d$.
- Efficacy: the dose demonstrates reasonable anti-disease activities, e.g., at least one “response" is observed at or below *b*.
- Sample size: The total cumulative number of patients at dose *b* (denoted as $n_b$, including patients from DE and backfill) satisfy $n_b \le n_{\text {cap}}$.

In the safety criterion, the second condition (i.e., $\hat{p}_{b}> \lambda _d$ but the pooled estimate $\hat{q}_{(b, b+1)} \le \lambda _d$) accounts for cases where additional data from backfill patients suggest that dose *b* might be overly toxic, exceeding the BOIN de-escalation boundary $\lambda _d$. However, the observed high DLT rate may occur sporadically due to the small sample size, as *b* is a lower dose and, therefore, should be safer than *c*. To reduce the likelihood of accidentally excluding a dose for backfill, the pooled estimate is used to determine whether the high DLT rate observed at *b* is reliable. As noted by Zhao et al. [9], the pooled estimate serves as an approximation to the isotonic estimate, offering nearly identical performance while being much simpler.

In the efficacy criterion, the term “response" is utilized in a generic sense to represent any clinically meaningful anti-disease activity. This criterion is established primarily based on ethical considerations, serving as a functional gatekeeper to ensure that backfilled patients are assigned only to dose levels demonstrating potential therapeutic activity. While definitive clinical efficacy (e.g., objective tumor response) is the preferred endpoint if observable early in the treatment cycle, many therapies require extended follow-up to assess these endpoints. In such instances, early surrogate biomarkers, such as target engagement, receptor occupancy, or other pharmacodynamic markers, may be employed to demonstrate promising biological activity and trigger eligibility for backfill accrual.

The above three criteria should be evaluated whenever a backfilling decision is being made. If the criteria are not met, backfilling should be paused for *b*. During the trial, it is possible for more than one dose to meet the backfill criteria. In such cases, different strategies can be employed to allocate patients. Patients can be randomized to all or a subset of backfill-eligible doses, or they can be assigned to the highest backfill-eligible dose, assuming that the efficacy of a higher dose is not lower than that of a lower dose.

The backfill eligibility criteria are structurally similar to those of BF-BOIN [9], but they differ in the calculation of $\hat{p}_b$ and $\hat{q}_{(b, b+1)}$. Here, the calculations incorporate pending patient data, whereas BF-BOIN uses only observed data. In BF-BOIN, DE is fully staggered before cohorts, so there are no pending patients in DE. However, backfill patients are enrolled without staggering, which means pending patients may be present when evaluating the backfill eligibility criteria. A common question we received regarding BF-BOIN is how ignoring pending backfill patients impacts operating characteristics. BE-BOIN provides a solution to this issue, which will be discussed in detail in the Simulation section.

#### Incorporating Backfill Data into DE

Backfilling provides additional data on doses previously cleared for safety by DE, which are based on a small number of patients (e.g., 3 patients). A challenge arises if additional patient data from backfilling results in a lower dose *b* having a higher toxicity rate than that of *c*. For example, if 0 out of 3 patients at *c* experience DLT, suggesting an escalation to *c+1*, this decision may conflict with data from a dose *b*, where 2 out of 5 patients experience DLT—-indicating a need to de-escalate at *b*. In this scenario, the additional DLTs at *b* are from backfilled patients (e.g., two backfilled patients at *b* experienced DLTs, while none of the three patients at *b* from DE experienced DLT). Table 1 summarizes potential data conflicts, defined as a situation where cumulative data at *b* suggests a more conservative decision than the data at *c*.

**Table 1:** Conflicting decisions between the current dose-escalation dose *c* and a backfilled dose *b*, where *c>b*

	Decision according to the data at current dose *c*		
Decision according to the data at a backfilled dose *b*	Escalate ($\hat{p}_c<\lambda _e$)	Stay ($\lambda _e<\hat{p}_c\le \lambda _d$)	De-escalate or Eliminate ($\hat{p}_c>\lambda _d$)
Escalate ($\hat{p}_b<\lambda _e$)	No conflict	No conflict	No conflict
Stay ($\lambda _e<\hat{p}_b\le \lambda _d$)	Conflict	No conflict	No conflict
De-escalate or Eliminate ($\hat{p}_b>\lambda _d$)	Conflict	Conflict	Conflict*

*This case does not necessarily mean that $\hat{p}_b> \hat{p}_c$. However, as $\hat{p}_b> \lambda _d$, it means that the additional data from backfilling patients demonstrate alarmingly higher toxicity than what originally observed during the dose escalation (i.e., $\hat{p}_b \le \lambda _e$). Therefore, it is important to reconcile such conflict for patient safety

When a data conflict occurs, it may be due to the randomness associated with the small sample size at *b*, or *b* may truly be toxic. In the latter case, some adjustment is needed for DE, noting that *c* is higher and thus should be more toxic than *b*, to ensure patient safety. Following a similar approach proposed by Zhao et al. [9], we pool data across doses to obtain a more reliable estimate of toxicity at *b*, facilitating such decision.

Specifically, let $b^*$ be the backfill dose (or the lowest dose when multiple doses are backfilled) whose data conflict with the data at the current dose *c* of DE. We calculate the pooled toxicity estimate $\hat{q}_{(b^*,c)}$ from $b^*$ to *c* using equation (2). The dose escalation/de-escalation rule in DE is adjusted as:

1. If $\hat{q}_{(b^*, c)} \le \lambda _e$, escalate to higher dose *c+1*.
2. If $\lambda _e < \hat{q}_{(b^*, c)} \le \lambda _d$, stay at the current dose *c*.
3. If $\hat{q}_{(b^*, c)}> \lambda _d$, de-escalate to the highest dose *k* that is deemed safe with $\hat{q}_{(b^*,k)} \le \lambda _d$, where $b^* \le k <c$. If such a dose does not exist, de-escalate to dose $b^*-1$.

Of note, if no data conflict is observed, no adjustment to DE is needed. One important advantage of using the pooled estimate is that the TITE-BOIN decision table provided in Supplementary Material S1 can still be used to make decisions of dose escalation or de-escalation. Supplementary Material S2 presents a hypothetical trial to demonstrate step-by-step implementation of BE-BOIN.

Again, the approach of identifying and resolving data conflict is similar to those of Zhao et al. [9], but they differ in the calculation of $\hat{q}_{(b^*, c)}$. Here, the calculations incorporate pending patient data, whereas BF-BOIN uses only observed data.

#### Determination of MTD and OBD

Upon the completion of DE and backfill, the MTD is selected based on the pooled data from DE and backfill patients. In BE-BOIN, we use the same dose selection rule as the BOIN: the MTD is selected as the dose whose DLT rate estimate, based on isotonic regression, is closest to the prespecified target *ϕ*. However, as noted by Liu and Yuan [22], DE and final MTD selection are two independent components, and BOIN is not restricted to isotonic regression. Any statistical model, such as a logistic model, can be fitted to the final data to provide the estimate of $p_j$ for MTD selection. We provide a simulation study in Supplementary Material S3 comparing isotonic and logistic regression for this purpose. The results align with established findings: as a nonparametric approach, isotonic regression yields robust performance across diverse dose-toxicity profiles. In contrast, logistic regression exhibits greater sensitivity to model specification. While it can offer enhanced precision when the underlying model is correctly specified, particularly when the MTD is at the highest dose level, it may yield inferior results when the dose-response relationship deviates from the assumed functional form.

After the identification of the MTD, a multiple dose randomization phase is often added to collect additional efficacy and toxicity data to better characterize the OBD. The doses for randomization are selected based on the totality of toxicity, efficacy, and pharmacokinetic and pharmacodynamic data from both DE and backfill patients. Often, the MTD ($d_{high}$) and one lower dose ($d_{low}$) are selected, and patients can be equally or adaptively randomized to these doses. Yang et al. [27] suggested that a sample size of 20-40 patients per dose arm often leads to reasonable accuracy in identifying the OBD.

Upon concluding the multiple-dose randomization phase, we employ a utility-based method [28–30] to select the OBD. This framework is highly flexible and scalable, facilitating a systematic integration of efficacy and toxicity. The approach begins by assigning utility scores to the four possible clinical outcomes: $\nu _1 =$ (efficacy, no toxicity), $\nu _2 =$ (efficacy, toxicity), $\nu _3 =$ (no efficacy, no toxicity), and $\nu _4 =$ (no efficacy, toxicity). We anchor these scores by assigning $\nu _1 = 100$ to the most favorable outcome and $\nu _4=0$ to the least favorable. Clinical experts are then tasked with assigning utility scores to the remaining intermediate outcomes, $\nu _2$ and $\nu _3$, reflecting the relative clinical desirability of these states. Let $(\pi _{j1}, \pi _{j2}, \pi _{j3}, \pi _{j4})$ represent the respective probabilities of the four outcomes at dose *j*. The estimated mean utility for dose *j* is defined as:

$$\begin{aligned} \hat{V}_j = \sum _{k=1}^{4} \nu _k \hat{\pi }_{jk}, \end{aligned}$$

where $\hat{\pi }_{jk}$ is the posterior mean estimate of $\pi _{jk}$ derived from a Dirichlet-multinomial model. Assuming a non-informative Dirichlet prior:

$$\begin{aligned} (\pi _{j1}, \pi _{j2}, \pi _{j3}, \pi _{j4}) \sim \text {Dirichlet}(\eta _1, \eta _2, \eta _3, \eta _4), \end{aligned}$$

with $\eta _1 = \eta _2 = \eta _3 = \eta _4 = 0.25$, corresponding to an effective prior sample size of one patient. Given the observed event counts $D_n = (n_{j1}, n_{j2}, n_{j3}, n_{j4})$ for each outcome at dose *j*, the posterior distribution is:

$$\begin{aligned} (\pi _{j1}, \pi _{j2}, \pi _{j3}, \pi _{j4}) \mid D_n \sim \text {Dirichlet}(\eta _1+n_{j1}, \eta _2+n_{j2}, \eta _3+n_{j3}, \eta _4+n_{j4}). \end{aligned}$$

The OBD is subsequently determined by comparing the posterior mean utilities, $\hat{V}_{d_{\text {high}}}$ and $\hat{V}_{d_{\text {low}}}$, and selecting the dose associated with the higher utility score.

Beyond utility-based frameworks, several other methods exist for determining the OBD. One common alternative is a marginal probability-based risk-benefit trade-off, such as $\hat{p}_{E, j} - w \cdot \hat{p}_{ j}$ [2]. Here, $\hat{p}_{E, j}$ and $\hat{p}_{j}$ denote the observed efficacy and toxicity rates for dose *j*, respectively, while *w* represents a pre-specified weight quantifying the trade-off between these two probabilities. Although this marginal approach appears straightforward, determining an appropriate value for *w* is often non-trivial. In practice, clinicians frequently find it more intuitive to assign utility scores to concrete, mutually exclusive clinical outcomes than to quantify an abstract trade-off weight across disparate probabilities. Consequently, we generally recommend the utility approach for its superior clinical interpretability. Furthermore, the utility framework is mathematically more general, as it encompasses the marginal probability approach as a special case–specifically when the utilities of the intermediate outcomes satisfy the condition $\nu _2 + \nu _3 = 100$ [28].

Alternatively, methods that implicitly account for the risk-benefit trade-off may be employed. For example, Wang et al. [31] proposed a simple decision rule: select $d_{\text {high}}$ if its efficacy rate exceeds that of $d_{\text {low}}$ by a pre-specified margin; otherwise, select $d_{\text {low}}$. While such rules are easy to implement and communicate, they are less flexible than utility-based methods because they do not allow for an explicit, multi-dimensional specification of the risk-benefit trade-off.

## Simulation Study

### Simulation Setting

We conducted a simulation study to evaluate the performance of the proposed BE-BOIN design. The target DLT rate *ϕ* was set at 0.25. Five dose levels were considered, with a total sample size *N* of 30 and a cohort size of 3. The sample size for backfill is capped at $n_{\text {cap}}=12$ at a dose. The DLT assessment window was set at three months, with an accrual rate of two patients per month. Statistically, the operating characteristics of a design are determined by the ratio of the DLT assessment window to the inter-patient arrival time (i.e., the reciprocal of the accrual rate), referred to as the A/I ratio, rather than the DLT window alone. For example, a one-month DLT assessment window with an accrual rate of six patients per month (fast accrual) yields the same operating characteristics as a three-month DLT window with an accrual rate of two patients per month (late-onset setting). The time to DLT event was sampled from Weibull distribution, with 50% of DLTs occurring in the latter half of the assessment window.

**Fig. 2:**
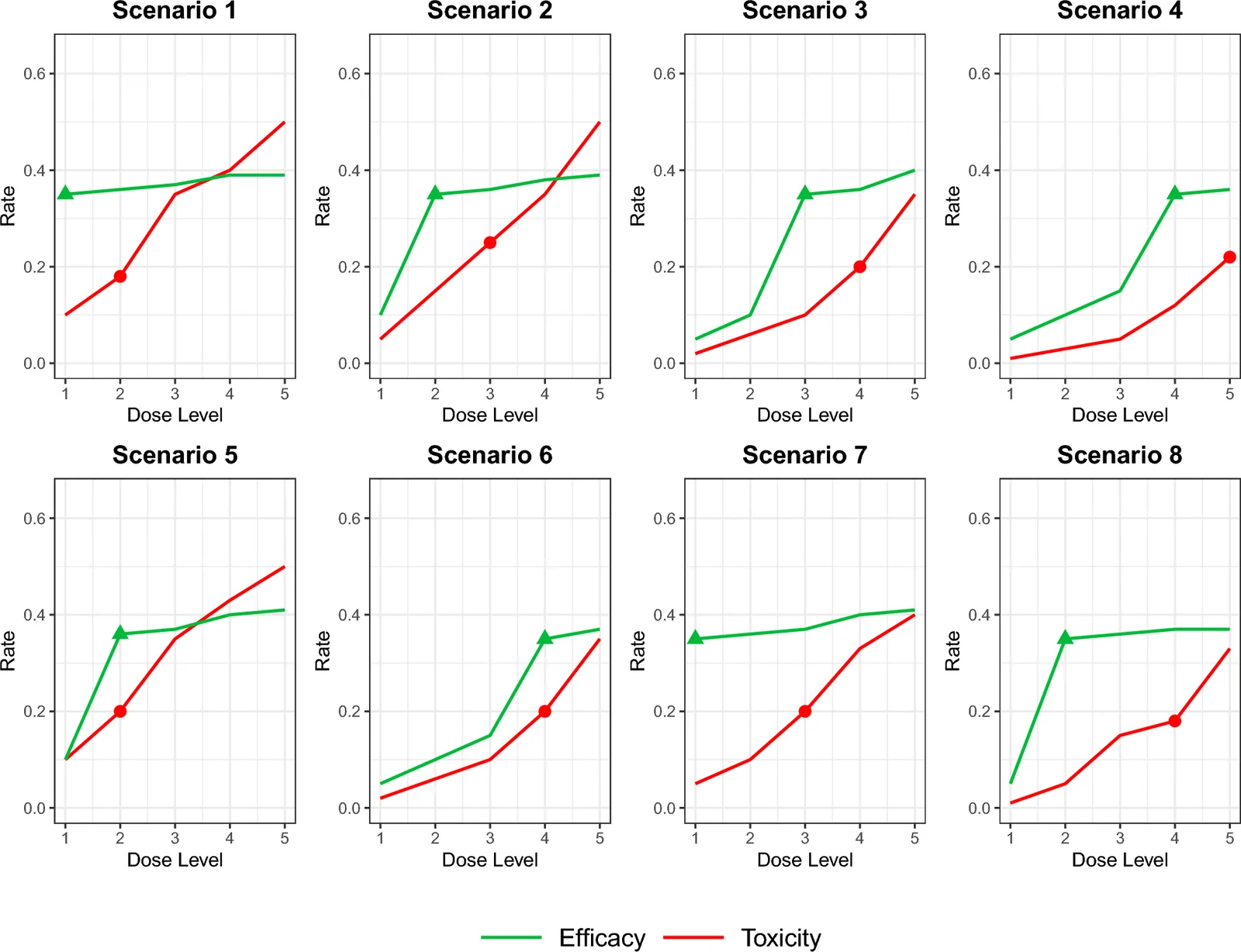
Plot of eight scenarios with different location of the maximum tolerated dose (MTD) and optimal biological dose (OBD), with MTD marked by circles and OBD indicated by triangles

After identifying the MTD in stage I, the trial proceeded to a multiple-dose randomized phase for dose optimization in stage II. Two doses, including MTD and the dose one level lower, were selected, with each receiving 20 additional randomly assigned patients. The utility scores $(\nu _1, \nu _2, \nu _3, \nu _4)$ were set as (100, 60, 40, 0). Simulations were conducted 10,000 times for each of the eight scenarios, representing various dose-toxicity and dose-response relationships, as depicted in Fig. 2. Specifically, in scenarios 1-4, the OBD was located one dose level below the MTD. In the scenarios 5-6, the OBD coincided with the MTD, and in scenarios 7-8, OBD was located two dose levels below the MTD.

We compared the BE-BOIN design to the TITE-BOIN design, which does not incorporate backfill, and the BF-BOIN design, which does not support real-time decision-making. Additionally, we compared BE-BOIN to the model-based backfill TITE-CRM, denoted as BE-CRM [6]. The BE-CRM model setting is presented in the Supplementary Material S4. To ensure a fair comparison and facilitate the interpretation of the results, the same accrual suspension rules and backfilling timing were used in BE-CRM and BE-BOIN.

Additionally, we compared BE-BOIN to BF-BOIN in the absence of late-onset toxicity by setting the DLT window to one month and the accrual rate of three patients per month. The DE cohorts are staggered to ensure that no pending data from DE patients occurs. The objective is to determine whether ignoring pending data from backfill patients, as done in BF-BOIN, impacts its operating characteristics compared to including pending data. We also conducted sensitivity analysis to evaluate the robustness of BE-BOIN. These analyses addressed the uniform time-to-event assumption, the impact of varying the A/I ratio from 3 to 9, and the effects of a high incidence of DLTs occurring later in the assessment window, such as over 50%.

The R Shiny application is under development, and the simulation code is available upon request.

### Simulation Results

Table 2 summarizes the simulation results comparing TITE-BOIN, BF-BOIN, BE-BOIN, and BE-CRM. After stage I, BE-BOIN achieves a robust accuracy of correct MTD selection(%), averaging 55.4% (range: 46.2–67.9), which exceeds TITE-BOIN’s 52.6% (range: 42.7–68.3) by an average of 2.7%. Compared to BF-BOIN, which leads with an average MTD selection of 56.1% (range: 44.5–68.8), BE-BOIN trails by only 0.7 percentage points. Across most scenarios, BE-BOIN outperforms BE-CRM, with an average improvement of 3.4% over BE-CRM’s 52.0% (range: 37.6–86.9). However, in scenario 4–where the MTD is the highest dose–BE-CRM achieves superior accuracy (86.9% vs. 67.9%), attributed to its tendency of aggressive escalation. On average, BE-CRM overdosed 3.9 more patients than BE-BOIN. For example, in scenarios 6 and 7, BE-BOIN overdosed 9.7 and 13.8 patients, respectively, while BE-CRM overdosed 16.5 and 19.4 patients.

**Table 2:** Operating characteristics of TITE-BOIN, BF-BOIN, BE-BOIN, and BE-CRM

Scenario	Method	MTD sel %	OBD sel %	Patients at MTD	Patients at OBD	Patients overdosed	Total patients	Trial duration
1	TITE-BOIN	53.9	45.9	28.6	21.6	17.8	68.0	49.1
	BF-BOIN	59.7	49.4	34.8	27.6	16.9	79.4	64.1
	BE-BOIN	56.9	47.7	32.6	26.7	17.0	76.3	47.2
	BE-CRM	53.2	43.9	33.5	23.4	18.9	75.8	45.5
2	TITE-BOIN	42.7	61.9	20.7	25.7	9.7	69.6	49.9
	BF-BOIN	48.2	67.7	23.9	32.3	7.8	80.3	64.4
	BE-BOIN	47.1	65.4	22.7	30.9	7.7	77.2	48.5
	BE-CRM	45.7	63.3	24.1	29.6	9.7	76.9	47.0
3	TITE-BOIN	54.1	53.8	24.8	21.4	10.7	70.0	52.4
	BF-BOIN	56.4	55.2	28.4	27.6	9.9	83.8	64.7
	BE-BOIN	57.4	57.4	26.7	26.9	9.4	80.3	51.0
	BE-CRM	44.3	40.8	28.6	20.7	16.9	80.7	50.0
4	TITE-BOIN	68.3	70.9	23.5	26.9	0.0	70.0	53.6
	BF-BOIN	68.8	71.2	23.5	32.6	0.0	85.4	64.9
	BE-BOIN	67.9	70.6	23.3	31.2	0.0	81.6	51.8
	BE-CRM	86.9	67.1	31.4	30.4	0.0	81.8	51.0
5	TITE-BOIN	55.5	73.1	28.5	28.5	16.4	67.6	49.0
	BF-BOIN	60.4	77.4	34.0	34.0	15.5	75.5	64.1
	BE-BOIN	58.8	76.6	32.1	32.1	15.4	73.0	47.2
	BE-CRM	56.2	76.6	33.6	33.6	16.5	72.2	45.3
6	TITE-BOIN	54.1	65.5	24.8	24.8	10.7	70.0	52.4
	BF-BOIN	56.2	66.6	28.4	28.4	9.8	82.7	64.7
	BE-BOIN	56.8	65.5	26.6	26.6	9.7	79.5	51.1
	BE-CRM	44.4	67.1	28.5	28.5	16.5	79.5	50.2
7	TITE-BOIN	49.5	10.5	25.0	8.3	15.4	69.9	50.8
	BF-BOIN	54.9	9.6	30.4	14.0	14.4	87.1	64.5
	BE-BOIN	51.9	10.5	28.2	13.5	13.8	82.6	49.2
	BE-CRM	47.5	9.0	28.6	10.9	19.4	83.1	48.3
8	TITE-BOIN	42.8	20.5	21.9	11.4	11.2	70.0	52.1
	BF-BOIN	44.5	21.3	25.2	17.9	10.2	86.4	64.7
	BE-BOIN	46.2	22.0	23.4	16.9	9.4	82.2	50.7
	BE-CRM	37.6	15.1	26.0	13.4	15.9	82.7	49.8

MTD sel % denotes the percentage of correct MTD selection after stage I, while OBD sel % denotes the percentage of correct OBD selection after stage II

After stage II, BE-BOIN maintains high accuracy in selecting the correct OBD across scenarios 1-6, averaging 63.9% (range: 47.7–76.6), which improves upon TITE-BOIN (61.8%, range: 45.9–73.1) by an average of 2.1 percentage. Compared to BF-BOIN, which achieves 64.6% (range: 49.4–77.4), BE-BOIN remains highly comparable with only a 0.7 percentage point difference. Notably, BE-BOIN outperforms BE-CRM in scenarios 1–4, where the true OBD lies one level below the MTD, with an average improvement of 6.5 percentage points, including sizable gains in scenario 3 (57.4% vs. 40.8%). In scenarios 5–6, where the OBD coincides with the MTD, BE-BOIN and BE-CRM are comparable. In contrast, all methods exhibit limited performance in scenarios 7–8, where the OBD is two dose levels below the MTD–resulting in correct OBD selection below 22%, due to stage II’s prioritization of MTD or MTD–1 doses.

One might initially be disappointed that BE-BOIN offers only modest improvement in correctly identifying the MTD and OBD compared to TITE-BOIN, despite treating, on average, 9.7 more patients. However, this result is anticipated: by design, backfilling tends to assign more patients to lower dose levels, which may contribute marginally to the precision of MTD and OBD identification. The primary objective of backfilling is to maximize patient enrollment within the same trial timeframe. This strategy not only expands patient access to novel therapies but also facilitates the collection of a far more comprehensive dataset, encompassing toxicity, efficacy, tolerability, pharmacokinetics/pharmacodynamics (PK/PD), biological activity, and various biomarkers. These data provide essential insights for guiding both OBD identification and subsequent clinical development decisions. Consequently, in practice, the actual gain in precision for OBD identification under the BE-BOIN design over TITE-BOIN is anticipated to be much more pronounced than demonstrated in this simulation study. We do not account for this in the current simulation because, while it is theoretically possible to specify a multi-dimensional rule that integrates these varied data types to assist in OBD identification, establishing a ’general’ rule that remains meaningful across diverse clinical settings and therapeutic areas is exceedingly difficult.

In practice, the choice between TITE-BOIN and BE-BOIN depends on the specific clinical context. If the primary goal is to identify the MTD with a minimal sample size, TITE-BOIN may be more appropriate. However, if robust dose optimization is the priority, particularly when accrual is rapid relative to the toxicity and efficacy evaluation windows, BE-BOIN is the preferable choice.

A major advantage of BE-BOIN over BF-BOIN is its substantially shorter trial duration. Leveraging real-time decision-making in stage I, BE-BOIN reduces trial duration by an average of 15.0 months (range: 13.1–16.9) compared to BF-BOIN’s 64.5 months, while maintaining comparable accuracy and safety. This reduction is primarily achieved during stage I, where BE-BOIN enables dose assignment for new patients with pending data, resulting in a 30–40% decrease in duration. In contrast, stage II–focused on multiple-dose randomized optimization–follows a fixed schedule, and all designs share the same duration.

Our simulation assumed a three-month DLT assessment window to clearly illustrate the impact of late-onset toxicity and the infeasibility of conducting a fully staggered phase I trial under such conditions (e.g., BF-BOIN would require approximately 64.5 months to complete). The advantage of the BE-BOIN design in reducing trial duration is not limited to settings with long DLT windows (e.g., three months); it is fully retained with shorter DLT windows (e.g., 28 days) and faster patient accrual. As noted in Section Simulation Setting, the operating characteristics of a design are primarily determined by the A/I ratio rather than by the DLT window alone.

### BE-BOIN vs BF-BOIN Without Late-Onset Toxicity

Table 3 compares the performance of BE-BOIN and BF-BOIN in the absence of late-onset toxicity. The objective is to evaluate whether ignoring pending backfill patients–as done in BF-BOIN–affects the operating characteristics of the design. Overall, the two designs yield nearly identical performance in terms of dose selection (including MTD identification) and patient allocation at each dose level, whether through the DE or BF components. Trial durations are also comparable. This similarity arises because, at each decision point, all patients in the DE component have complete follow-up, and DE primarily guides dose decisions. These results support the validity of the BF-BOIN approach in settings without late-onset toxicity.

**Table 3:** Comparison between BE-BOIN and BF-BOIN design in settings without late-onset toxicity

Scenario	Method	Metrics	*d*1	*d*2	*d*3	*d*4	*d*5	Trial duration
1	BF-BOIN	Sel(%)	7.93	**58**.**15**	28.87	4.27	0.54	17.97
		Patients	13.01	**15**.**77**	7.82	1.67	0.27	
	BE-BOIN	Sel(%)	8.40	**57**.**54**	29.12	4.21	0.45	18.01
		Patients	13.22	**15**.**65**	7.67	1.65	0.25	
2	BF-BOIN	Sel(%)	2.13	34.44	**45**.**89**	15.71	1.80	18.08
		Patients	8.45	14.27	**10**.**60**	3.96	0.80	
	BE-BOIN	Sel(%)	2.16	34.99	**45**.**66**	15.35	1.82	18.09
		Patients	8.43	14.39	**10**.**53**	3.91	0.76	
3	BF-BOIN	Sel(%)	0.04	1.88	16.47	**54**.**94**	26.67	18.21
		Patients	4.72	7.58	11.74	**11**.**08**	4.88	
	BE-BOIN	Sel(%)	0.08	2.05	15.87	**55**.**92**	26.08	18.22
		Patients	4.71	7.59	11.80	**11**.**08**	4.90	
4	BF-BOIN	Sel(%)	0.00	0.18	2.57	27.23	**70**.**02**	18.30
		Patients	4.03	5.95	9.30	12.12	**9**.**81**	
	BE-BOIN	Sel(%)	0.00	0.26	2.44	27.23	**70**.**07**	18.32
		Patients	4.02	5.90	9.22	12.14	**9**.**86**	
5	BF-BOIN	Sel(%)	10.81	**59**.**58**	25.77	3.26	0.33	17.99
		Patients	11.36	**15**.**40**	7.07	1.46	0.20	
	BE-BOIN	Sel(%)	10.96	**59**.**67**	25.27	3.34	0.50	17.97
		Patients	11.45	**15**.**27**	7.04	1.47	0.22	
6	BF-BOIN	Sel(%)	0.08	2.04	16.00	**55**.**55**	26.33	18.22
		Patients	4.71	7.59	10.91	**11**.**01**	4.92	
	BE-BOIN	Sel(%)	0.04	2.09	16.01	**55**.**35**	26.51	18.20
		Patients	4.70	7.56	10.88	**10**.**94**	4.87	
7	BF-BOIN	Sel(%)	0.51	14.69	**52**.**86**	27.15	4.77	18.13
		Patients	9.77	13.25	**12**.**64**	5.96	1.39	
	BE-BOIN	Sel(%)	0.51	15.10	**52**.**87**	26.35	5.14	18.12
		Patients	9.83	13.25	**12**.**61**	5.97	1.42	
8	BF-BOIN	Sel(%)	0.05	2.94	25.10	**42**.**82**	29.09	18.21
		Patients	4.54	10.44	12.30	**9**.**80**	4.80	
	BE-BOIN	Sel(%)	0.04	3.16	24.20	**44**.**15**	28.45	18.20
		Patients	4.55	10.44	12.23	**9**.**83**	4.84	

sel % denotes the percentage of selecting each dose as the MTD after stage I. Patients represent the average number of patients treated at each dose

### Sensitivity Analysis

In Supplementary Material S5, Figure A2 presents sensitivity analysis based on data generated from Log-logistic, Uniform, and Weibull time-to-event distributions. BE-BOIN exhibits consistent performance across all distributions, underscoring its robustness to the underlying time-to-event assumptions. Figure A3 shows results under varying A/I ratios, with the fixed DLT assessment window of 3 months: A/I = 3 (one patient/month), A/I = 6 (two patients/month), and A/I = 9 (three patients/month). Higher A/I ratios lead to greater reductions in trial duration, more patients treated at the MTD and OBD, and fewer overdosed patients. Nonetheless, differences in operating characteristics across A/I settings remain minor. Figure A4 reports results using Weibull-distributed time-to-event data with varying proportions of late-onset toxicities–defined as DLTs occurring in the second half of the assessment window: 30%, 50%, and 70%. As the proportion of late-onset toxicity increases, the number of overdosed patients decreases. This is because a higher proportion of late-onset toxicity results in less data being available for real-time decision-making. BE-BOIN appropriately accounts for this uncertainty by adopting a more conservative dose-escalation strategy, leading to fewer overdosed patients. Notably, the trial duration remains largely unaffected by the timing of DLT onset.

## Conclusion

We propose the BE-BOIN design, which allows backfilling patients into safe and effective doses during dose escalation and accommodates late-onset toxicities. BE-BOIN enables the collection of additional safety and efficacy data to enhance the accuracy and reliability of optimal dose selection and supports real-time dose decisions for new patients. Our simulation studies show that BE-BOIN accurately identifies the MTD/OBD while significantly reducing trial duration. As a byproduct, our results also demonstrate that the BF-BOIN approach of ignoring pending backfilling patients has a negligible impact on the operating characteristics of the design, addressing a common question raised about BF-BOIN.

Following TITE-BOIN, the default assumption of BE-BOIN is that the time to toxicity follows a uniform distribution over the DLT window. This assumption is adopted for simplicity and because the design has been shown to be remarkably robust to its violation, as discussed previously. However, this assumption is not required. A more flexible piecewise uniform distribution can be used–for example, by partitioning the DLT window into several intervals (e.g., three) and assuming a uniform distribution within each interval. If DLTs are more likely to occur later in the assessment window (e.g., under frequent but lower-toxicity dosing regimens), a prior distribution assigning greater weight to the later portion of the window can be specified. Conversely, if early-onset DLTs are expected (e.g., under less frequent but higher-dose regimens), more weight can be assigned to the earlier portion of the window [20]. The TITE-BOIN software provides this functionality and can generate the corresponding decision table for use in BE-BOIN trials.

To align with common practice in phase I trials, we assume that the DLT window is prespecified and sufficiently wide to capture all DLTs relevant to dose escalation and selection. However, our methodology itself does not rely on this assumption. For example, a trial protocol may prespecify a 21-day DLT window corresponding to the first treatment cycle. During the trial, if DLTs unexpectedly occur outside the prespecified window, it is advisable to extend the DLT window through a protocol amendment to incorporate these events into decision-making. Once the data are updated according to the revised window, the BE-BOIN design can proceed without modification. This robustness arises from a key feature inherited from TITE-BOIN–its decision table depends only on the standardized total follow-up times (relative to the DLT window), making it inherently independent of the specific window length (see Supplementary Material S1 for an example). The primary challenge in modifying the DLT window during the trial lies in simulating the operating characteristics of the design. The rule that triggers the modification must be fully specified, and additional assumptions (e.g., the distribution of DLTs occurring outside the initial DLT window) are required to develop simulation software capable of evaluating the design’s operating characteristics.

BE-BOIN employs a two-stage design framework: Stage 1 performs dose escalation to identify the MTD, after which two or more doses are selected for Stage 2 randomization and optimization. However, this framework inherits a limitation of two-stage designs: if the initial doses selected for Stage 2 do not include the OBD, which is rather likely given the small sample sizes in Stage 1, the design will fail to identify the OBD, regardless of the increased patient enrollment in Stage 2. To address this limitation, one can employ an efficacy-integrated design framework [2], where the OBD estimate is continuously updated as data accumulate throughout the trial. For instance, observed data can be used to perpetually refine utility estimates to guide dose escalation decisions. Such continuous learning enables more efficient and robust identification of the OBD. Nevertheless, the efficacy-integrated approach has its own constraints. Most notably, it requires the continuous observation of efficacy data. While a TITE-BOIN-type approach can be used to manage pending efficacy outcomes, estimates may still exhibit high variability due to limited initial data. Furthermore, efficacy-integrated designs typically assume a homogeneous population. In contrast, the two-stage approach offers greater flexibility: it allows for dose escalation in an "all-comers" population during Stage 1, while focusing Stage 2 dose optimization on a specific subgroup or indication.

Recently, Takeda et al. [32] proposed the BF-BOIN-ET design, which also accommodates backfilling with delayed outcomes. While both BE-BOIN and BF-BOIN-ET share the goal of integrating backfilling with real-time decision-making under pending data, they differ in purpose and in practice. BE-BOIN is based on the original BOIN design and aims to identify the MTD; dose escalation and de-escalation decisions are driven primarily by toxicity data. Efficacy data observed from lower doses are used only to ensure that backfilled patients are assigned to beneficial doses, and are not incorporated into dose-level decisions. In contrast, the BF-BOIN-ET design is based on the BOIN-ET framework, which aims to identify the optimal dose using both efficacy and toxicity data, and both types of data are required to inform dose decisions. When the efficacy endpoint assessment window is substantially longer than the DLT assessment window (e.g., 12 weeks versus 4 weeks), BF-BOIN-ET would require frequent suspension of enrollment until sufficient data at the current dose are available to support a decision. In this setting, BE-BOIN offers a practical advantage, as efficacy data at the current dose are not needed for decision-making. On the other hand, when the efficacy assessment window is comparable to the DLT window, BF-BOIN-ET has the advantage of early detection of the optimal dose and higher optimal dose selection, particularly when it is far away from the MTD. A detailed comparison of the two designs is provided in Supplementary Material S6.

## Supplementary Information

Below is the link to the electronic supplementary material.

Supplementary file 1

## Data Availability

No datasets were generated or analysed during the current study.
