# Supplementary material for: BE-BOIN: A Dose Optimization Design Accommodating Backfill and Late-Onset Toxicity
Source: Ther Innov Regul Sci. 2026 Aug 10;60(6):1848–60. doi: 10.1007/s43441-026-00994-0 (PMC13722620; doi:10.1007/s43441-026-00994-0)
Supplement: Supplementary file 1 [file 43441_2026_994_MOESM1_ESM.pdf]

Supplementary Materials for "BE-BOIN: A Dose Optimization Design Accommodating Backfill and Late-Onset Toxicity"

Kai Chen[1], **Yixuan Zhao**[2], **Kentaro Takeda**[3], **Ying Yuan**[4,*]

[1]Eli Lilly and Company, Indianapolis, IN, USA

[2]Department of Biostatistics and Data Science, The University of Texas Health Science Center, Houston, TX

[3]Astellas Pharma Global Development Inc., Northbrook, IL, USA

[4]Department of Biostatistics, The University of Texas MD Anderson Cancer Center, Houston, TX

[*]Author for correspondence: yyuan@mdanderson.org

S1 Decision table

We utilize the same TITE model and suspension rules as in the TITE-BOIN design, allowing the decision table to be directly applied in the BE-BOIN framework. During the trial, at each decision point, the following data are recorded for each dose: the total number of patients, the number of observed DLTs, the number of pending patients, and two key metrics—TF (Standardized Total Follow-Up Time) and MF (Standardized Minimum Follow-Up Time). TF represents the cumulative follow-up time of all pending patients divided by the DLT assessment window, while MF is calculated as the minimum follow-up time among all pending patients divided by the DLT assessment window. These metrics ensure consistency and reliability in decision-making.

Table A1: Dose-escalation and de-escalation boundaries up to 9 patients, with a target DLT rate of 0.25 and cohort size of 3. The cutoff A in suspension rule 1 is 51 %, and the cutoff B in suspension rule 2 is 25 %.

**No. patients**	**No. DLT**	**No. pending**	**Suspension**	**Escalation**	**Stay**	**De-escalation**
3	0	0	No	Yes	No	No
3	0	1	MF $<$ 0.25	MF $\geq$ 0.25	No	No
3	0	$\geq$ 2	Yes	No	No	No
3	1, 2	$\leq$ 2	No	No	No	Yes
3	3	0	No	No	No	Yes & Eliminate
6	0	0	No	Yes	No	No
6	0	1, 2	MF $<$ 0.25	MF $\geq$ 0.25	No	No
6	0	$\geq$ 3	Yes	No	No	No
6	1	0	No	Yes	No	No
6	1	1	MF $<$ 0.25 & TF $\geq$ 0.22	MF $\geq$ 0.25 & TF $\geq$ 0.22	TF $<$ 0.22	No
6	1	2	MF $<$ 0.25 & TF $\geq$ 1.38	MF $\geq$ 0.25 & TF $\geq$ 1.38	TF $<$ 1.38	No
6	1	$\geq$ 3	Yes	No	No	No
6	2, 3	$\leq$ 4	No	No	No	Yes
6	$\geq$ 4	$\leq$ 2	No	No	No	Yes & Eliminate
9	0	0	No	Yes	No	No
9	0	1–4	MF $<$ 0.25	MF $\geq$ 0.25	No	No
9	0	$\geq$ 5	Yes	No	No	No
9	1	0	No	Yes	No	No
9	1	1–3	MF $<$ 0.25	MF $\geq$ 0.25	No	No
9	1	4	MF $<$ 0.25 & TF $\geq$ 0.66	MF $\geq$ 0.25 & TF $\geq$ 0.66	TF $<$ 0.66	No
9	1	$\geq$ 5	Yes	No	No	No
9	2	$\leq$ 4	No	No	Yes	No
9	2	$\geq$ 5	Yes	No	No	No
9	3, 4	$\leq$ 6	No	No	No	Yes
9	$\geq$ 5	$\leq$ 4	No	No	No	Yes & Eliminate

Note: "No. patients" is the total number of patients treated at one dose. "No. DLT" is the number of patients who experienced DLT at one dose. "No. pending" denotes the number of patients whose DLT data are pending. TF: standardized total follow-up time of pending patients. MF: standardized minimum follow-up time of pending patients. When a dose is eliminated, all higher doses should also be eliminated.

S2 Hypothetical trial example

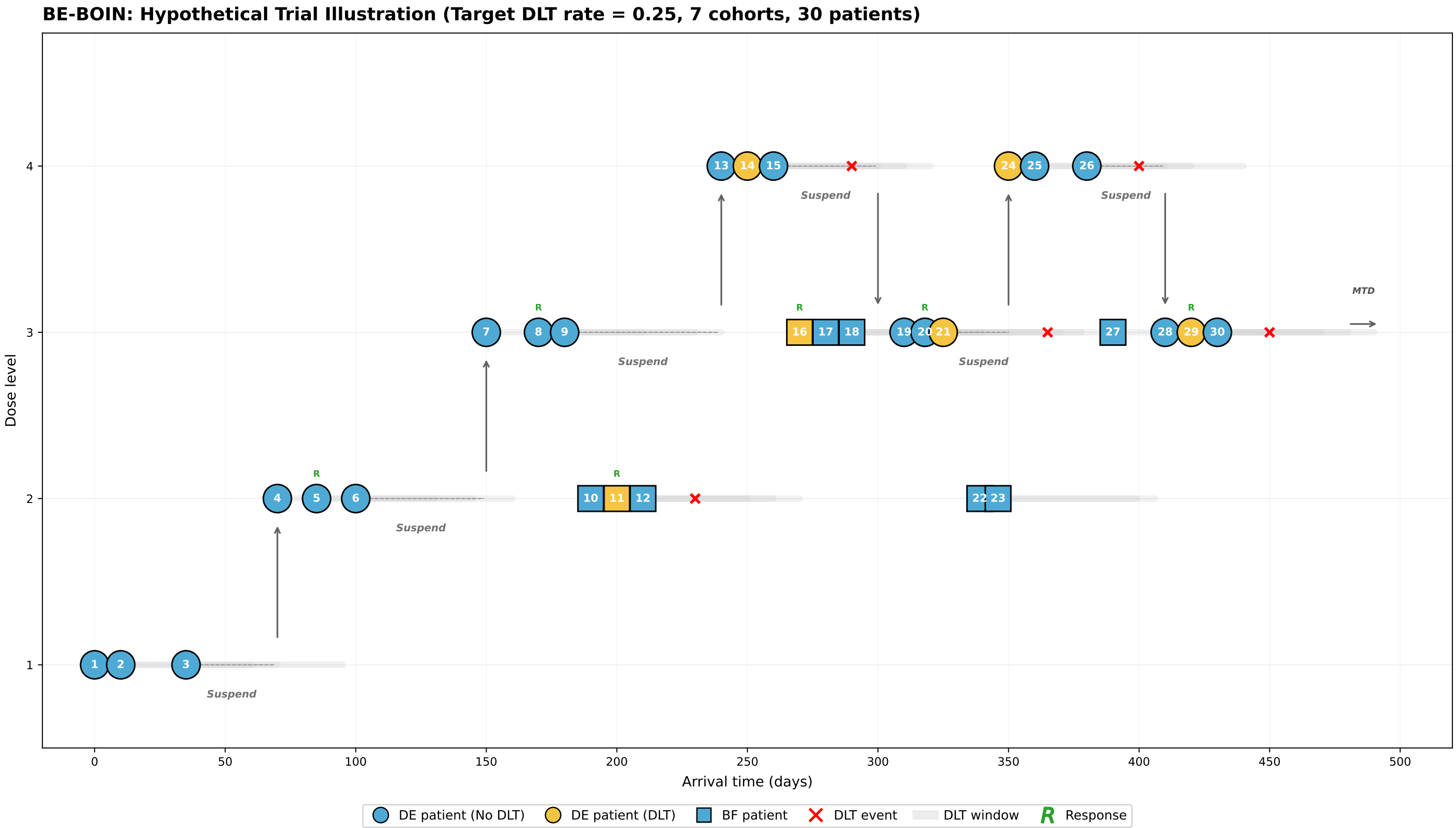

Figure A1: A hypothetical trial example of BE-BOIN using a target DLT rate = 0.25, with dose escalation performed in cohorts of 3 patients, and 7 cohorts. The cutoff A in suspension rule 1 is 51 %, and the cutoff B in suspension rule 2 is 25 %. At the end of the trial, the DLT results from all 30 patients (9 of which were backfilled) are used to estimate the MTD to be dose level 3.

To illustrate the BE-BOIN design, consider a trial with a target DLT rate of $\phi = 0.25$, seven cohorts of three patients each, and a 60-day DLT evaluation window. The implementation is depicted in Figure A1 and detailed as follows:

The first cohort of three patients (patients 1–3) are treated at dose level 1 (arrival days 0, 10, 35). According to Rule 1 and Rule 2, accrual is suspended until the first 2 patients finish the DLT assessment (at day 70) and the third patient has been followed for 25% of the DLT window. Following the BE-BOIN rule, the suspension is lifted and the second cohort (patients 4–6) is treated at dose level 2. By day 145, patients 4 and 5 complete the DLT window without DLT and patient 5 has a response observed; patient 6 has completed 25% of the DLT window. The suspension is lifted, and we treat the next cohort (patients 7–9) at dose level 3. Before patients 7 and 8 complete the DLT window at day 230, three patients (patients 10–12) arrived at days 190, 200, and 210 and were backfilled to dose level 2. By the time patient 13 arrived at day 240, the cohort 2 at dose level 2 had completed assessment with 0/3 DLTs, while among the

backfill patients (10–12) at dose level 2, patient 10 had one DLT event. According to the BE-BOIN decision table A1 in S2 (6 patients, 1 DLT, 2 pending, TF = $(40+30)/60 < 1.38$), a stay decision could be made for dose level 2, while at dose level 3 a dose escalation could be made (DLT rate = 0/3). Per Table 1 in the main text, a "stay versus escalate" conflict was observed between dose levels 2 and 3. Accordingly, data from dose 2 to dose 3 were pooled ($6 + 3 = 9$ patients), and following the decision table (9 patients, 1 DLT, 2 pending, TF = $30/60 = 0.5$), the dose was escalated to level 4, and dose level 3 was opened for backfilling. Subsequently, patients 13–15 (arrived at days 240, 250, 260) were assigned to dose level 4 as the fourth cohort in DE. During the follow-up of patients at dose level 4, patients 16–18 arrived (days 270, 280, 290) and were backfilled to dose level 3. At day 300, patient 14's DLT was observed, and the overall observed DLT rate at dose level 4 was at least 1/3; a de-escalation was made. Patients 19–21 (arrived at days 300, 310, 320) were then treated at dose level 3 as the fifth cohort in DE. Before day 350, DE was suspended and additional patients 22–23 arrived at days 330 and 335 and were backfilled to dose level 2. At day 350, 9 patients had been treated at dose level 3, with 6 patients having completed the DLT window with 1 DLT observed and 3 pending patients with MF = 0.33. An escalation decision was made and patients 24–26 (arrived at days 350, 360, 380) were treated at dose level 4 as the sixth cohort in DE. At day 390, patient 27 arrived, and at this time all patients at dose level 3 had completed the DLT assessment with 2 DLTs observed ($2/9 = 0.22$); patient 27 was backfilled to dose level 3. At day 400, one more DLT was observed at dose level 4 and a de-escalation was made again. Newly arrived patients 28–30 (arrived after day 400) were treated at dose level 3 as the seventh cohort in DE. The pre-specified 7 cohorts of patients for DE was reached, enrollment stopped, and an isotonic regression using all the data identified dose level 3 as the MTD.

S3 Comparison of isotonic regression and logistic regression for MTD selection

Table A2 compares isotonic regression and logistic regression for MTD selection at the end of stage I. Scenarios 1–4 use the same toxicity probabilities as in the main simulation study, where the MTD is located at doses 2, 3, 4, and 5, respectively, and the dose spacing is irregular (i.e., the toxicity probabilities are not generated from a logistic model). Scenarios 9–12 use toxicity probabilities generated from a logistic dose-toxicity model with the MTD at the same dose locations. In Scenarios 1–4, isotonic regression achieves higher correct MTD selection than logistic regression when the MTD is located at lower or intermediate doses (Scenarios 1–3), with the largest difference observed in Scenario 3 (53.4% vs. 43.4%). In contrast, when the MTD is located at the highest dose (Scenario 4), logistic regression substantially outperforms isotonic regression (77.5% vs. 66.8%). A consistent pattern across Scenarios 1–4 is that logistic regression tends to select overly toxic doses; for example, in Scenario 3, the selection of overly toxic doses (doses above the MTD) increases from 23.4% under isotonic regression to 35.4% under logistic regression. This tendency may be attributable to the parametric logistic model imposing a smooth monotone curve, which can oversmooth the dose-toxicity relationship when the true curve does not conform to the assumed logistic shape, leading to underestimation of toxicity at higher doses and a greater likelihood of selecting an overly toxic dose as the MTD.

In Scenarios 9–12, where the true dose-toxicity relationship follows a logistic model, the two methods yield more comparable results. When the MTD is at the highest dose (Scenario 12), logistic regression again demonstrates a clear advantage (48.5% vs. 36.8%), as the parametric model effectively borrows information across doses and extrapolates the dose-toxicity curve to the upper end where data are sparse. Overall, these results suggest that isotonic regression is a robust default for MTD selection across a wide range of dose-toxicity shapes, while logistic regression offers a meaningful improvement specifically when the MTD resides at the highest dose level and data at that dose are limited.

Table A2: Comparison of isotonic regression and logistic regression for MTD selection.

Scenario	Method	Metrics	d_1	d_2	d_3	d_4	d_5
1		p_T	0.10	<u>0.18</u>	0.35	0.40	0.50
	Isotonic	Sel(%)	15.95	**55.25**	24.65	3.90	0.15
		Patients	11.28	11.75	5.83	1.23	0.61
	Logistic	Sel(%)	14.20	**49.00**	30.60	5.50	0.50
		Patients	11.32	11.62	5.90	1.28	0.58
2		p_T	0.05	0.15	<u>0.25</u>	0.35	0.50
	Isotonic	Sel(%)	4.35	36.15	**43.80**	14.65	1.05
		Patients	7.90	12.34	8.71	3.01	1.03
	Logistic	Sel(%)	3.90	31.45	**43.95**	18.40	2.30
		Patients	7.91	12.34	8.51	2.87	0.92
3		p_T	0.02	0.06	0.10	<u>0.20</u>	0.35
	Isotonic	Sel(%)	0.60	5.15	17.45	**53.40**	23.40
		Patients	5.02	7.67	10.67	9.62	4.60
	Logistic	Sel(%)	0.70	3.70	16.80	**43.40**	35.40
		Patients	4.85	7.54	10.88	9.51	4.30
4		p_T	0.01	0.03	0.05	0.12	<u>0.22</u>
	Isotonic	Sel(%)	0.05	1.25	4.75	27.20	**66.75**
		Patients	4.37	6.44	8.90	11.01	8.11
	Logistic	Sel(%)	0.05	1.10	3.20	18.20	**77.45**
		Patients	4.34	6.37	8.80	11.28	8.41
9		p_T	0.08	<u>0.25</u>	0.43	0.57	0.68
	Isotonic	Sel(%)	21.15	**67.00**	11.25	0.60	0.00
		Patients	12.55	12.29	4.22	0.69	0.35
	Logistic	Sel(%)	16.45	**63.95**	18.80	0.80	0.00
		Patients	12.48	12.42	4.25	0.63	0.37
10		p_T	0.04	0.13	<u>0.25</u>	0.37	0.48
	Isotonic	Sel(%)	3.10	32.10	**49.90**	13.80	1.10
		Patients	7.43	12.38	9.27	3.12	0.99
	Logistic	Sel(%)	2.70	26.20	**48.20**	20.05	2.85
		Patients	7.40	12.41	9.46	3.21	0.99
11		p_T	0.02	0.08	0.16	<u>0.25</u>	0.34
	Isotonic	Sel(%)	0.50	10.00	36.35	**39.95**	13.20
		Patients	5.13	8.84	10.97	7.22	2.97
	Logistic	Sel(%)	0.50	8.00	30.25	**40.10**	21.15
		Patients	5.15	8.82	10.91	7.14	2.81
12		p_T	0.01	0.05	0.11	0.18	<u>0.25</u>
	Isotonic	Sel(%)	0.20	2.95	20.30	39.80	**36.75**
		Patients	4.63	7.76	10.16	9.20	5.08
	Logistic	Sel(%)	0.15	2.70	15.30	33.35	**48.50**
		Patients	4.60	7.59	10.06	9.29	5.29

Sel(%) denotes the percentage of selecting each dose as the MTD. Patients represent the average number of patients treated at each dose. The true MTD (underlined in p_T row) is the dose with toxicity probability closest to the target rate of 0.25.

S4 Model setting for BE-CRM design

Barnett et al. (2023) employ a two-parameter logistic model, defined as:

$$p_j = \frac{\exp\left(a_0 + \beta d_j^*\right)}{1 + \exp\left(a_0 + \beta d_j^*\right)}, \quad j = 1, \ldots, J,$$

where d_j^* represents the standardized dose at level j. In the simulations, we adjusted d_j^* to set the prior toxicity rate skeleton as (0.01, 0.10, 0.25, 0.45, 0.60), positioning the target DLT rate in the middle. The prior of a_0 is $N(\log(16), 4)$, and prior of β is $N(0, 1)$.

Following the methodology described in the original paper (Barnett et al., 2023), two early stop rules are implemented for safety. The first rule stops the trial if the lowest dose is deemed too toxic, defined by:

$$\Pr(p_1 > \phi_T | D) > \eta,$$

where η is set at 0.80 for the simulations. The second rule excludes a dose, and all higher doses, from further experimentation if the probability that the toxicity exceeds the target toxicity is high, specified as:

$$\Pr(p_j > \phi_T | D) > 0.95.$$

This second safety criterion relies on a beta-binomial model with a Beta(1,1) prior.

At the end of trial, the same logistic model is fitted based on all cumulative data. The MTD is selected from the dose having the toxicity rate closest to target DLT rate.

S5 Results of sensitivity analysis

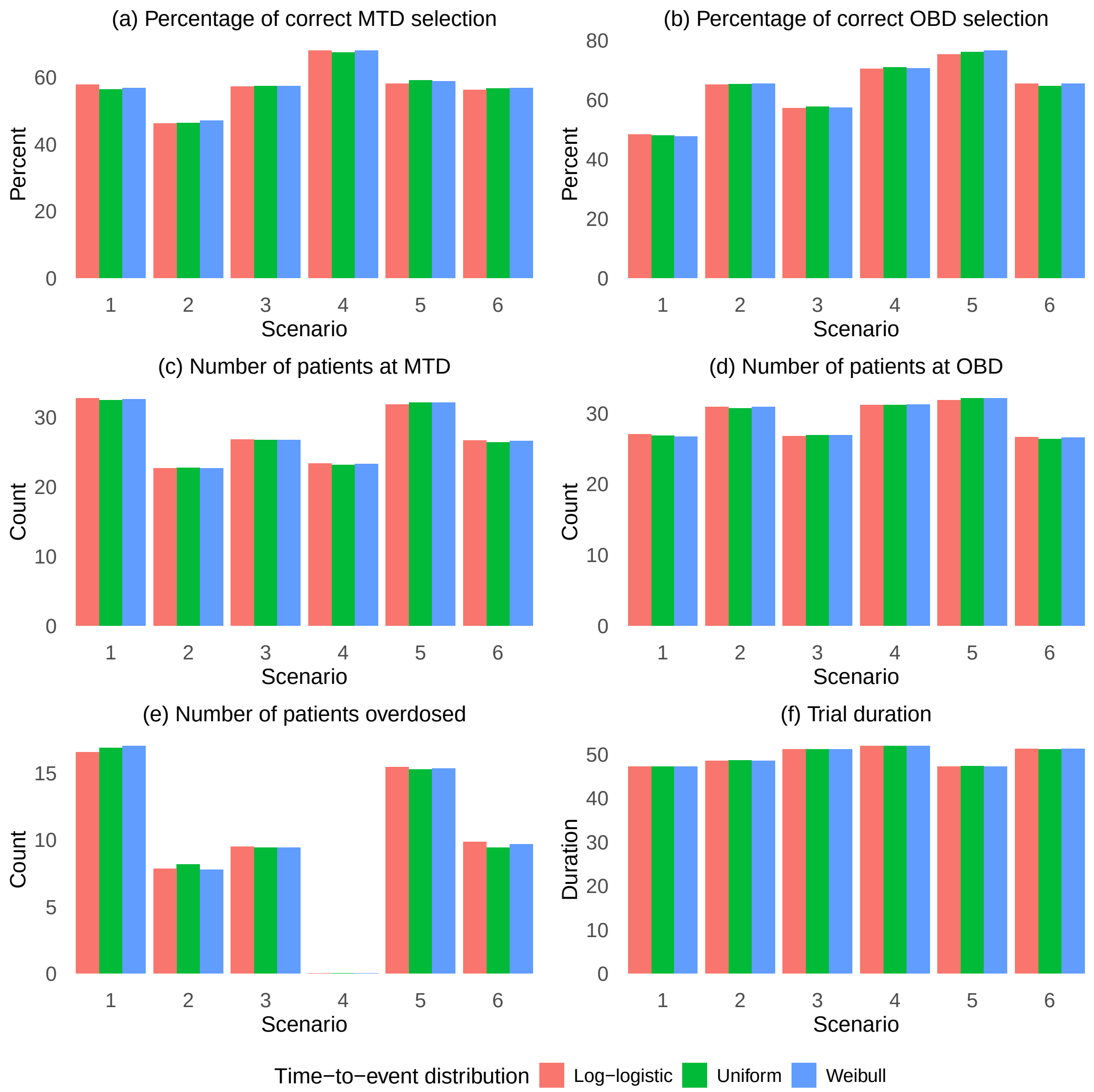

Figure A2: Sensitivity analysis of BE-BOIN design under different time-to-event distribution, including Weibull, Uniform and Log-logistic distribution.

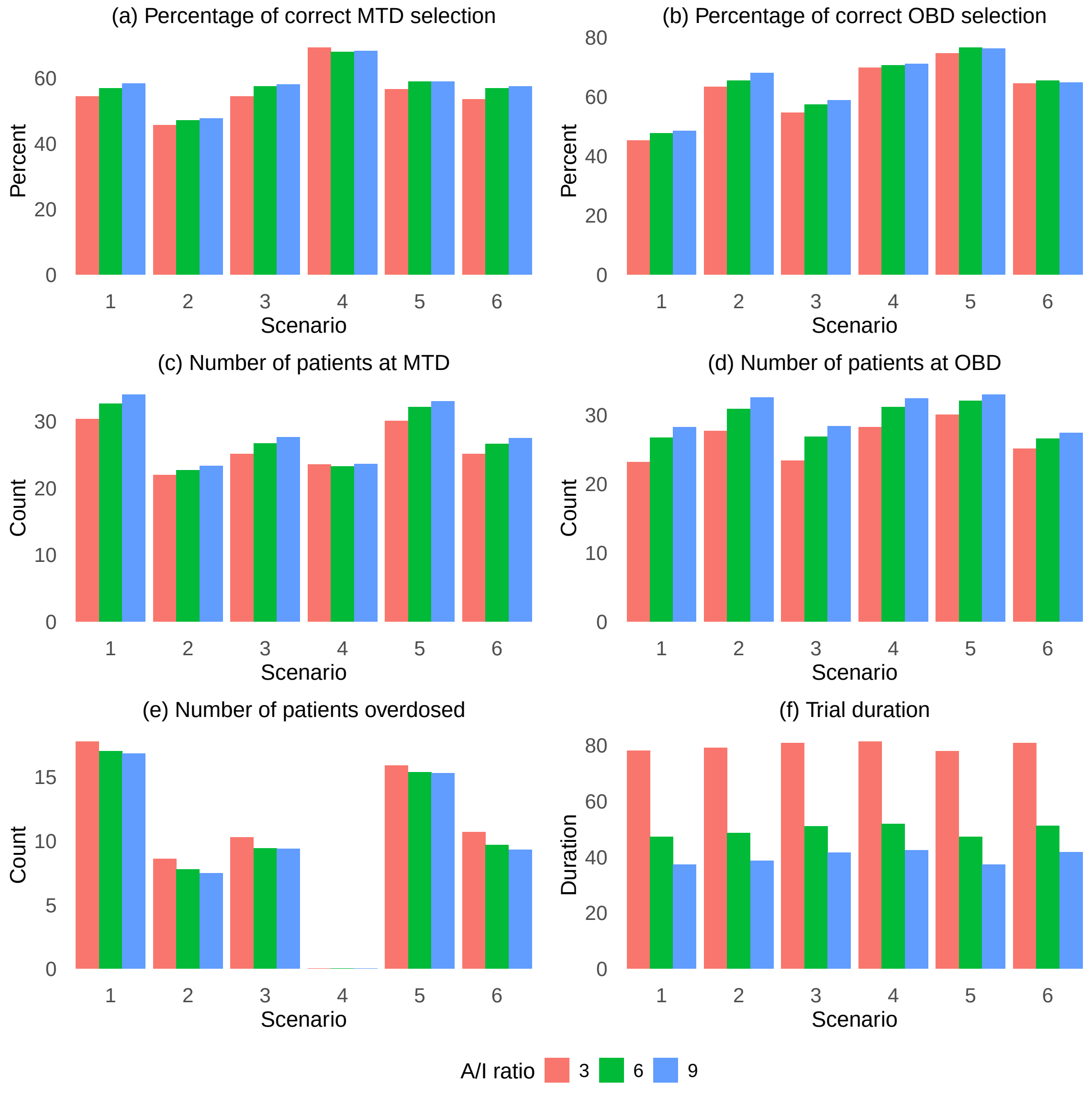

Figure A3: Sensitivity analysis of BE-BOIN design under different A/I ratios.

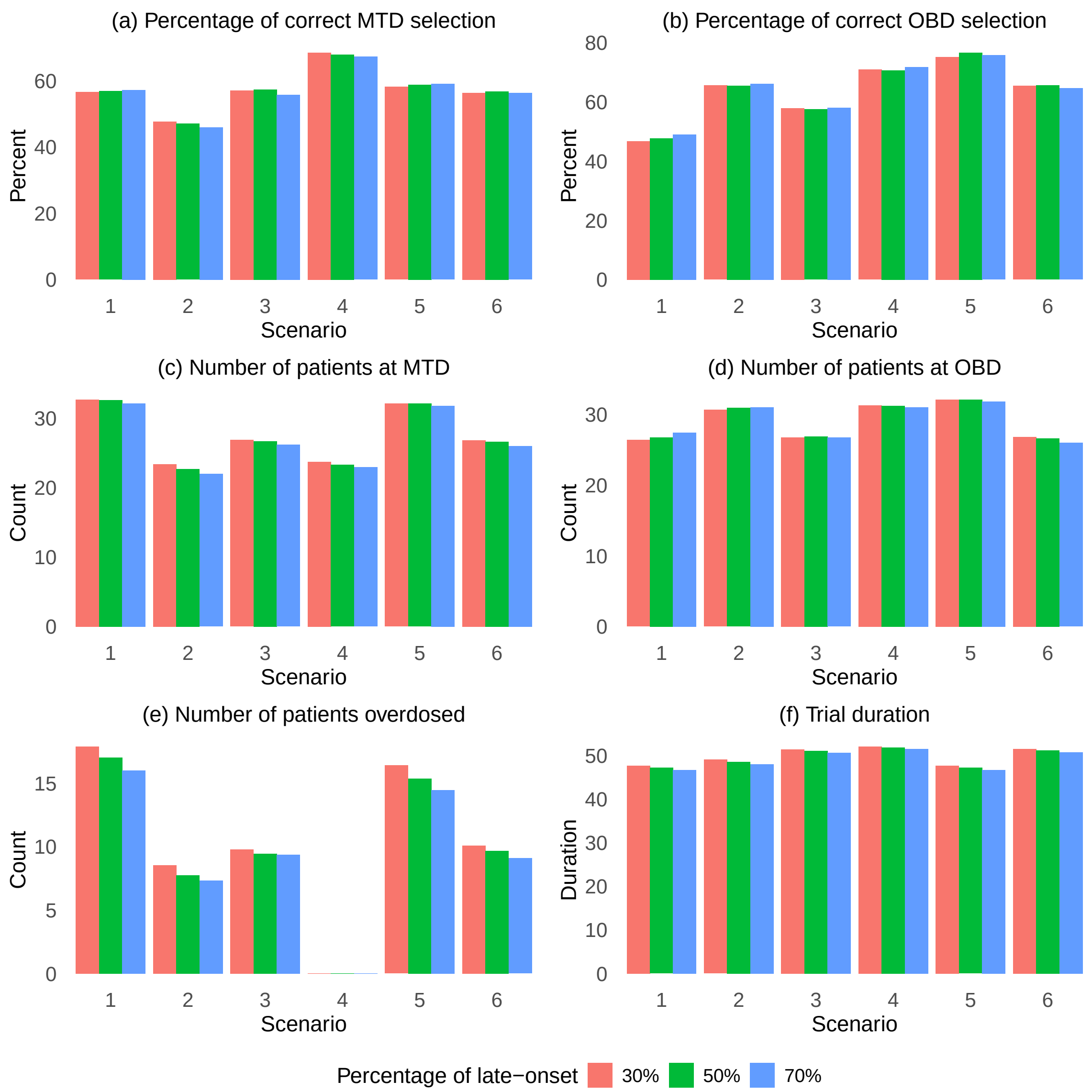

Figure A4: Sensitivity analysis of BE-BOIN design under different late-onset profiles for toxicity.

S6 BE-BOIN vs BF-BOIN-ET

Table A3 presents the comparison of BE-BOIN and BF-BOIN-ET across eight scenarios. For both designs, the OBD is selected at the end of stage I using the utility-based approach described in Section 2.2.4. Overall, the two designs yield similar performance across key operating characteristics. BE-BOIN tends to achieve slightly higher MTD selection accuracy in most scenarios, while BF-BOIN-ET shows a modest advantage in OBD selection in several scenarios. These differences are generally within 2 to 6 percentage points, suggesting that both designs are competitive for dose identification.

BE-BOIN has slightly more total patients than BF-BOIN-ET design. Despite this modest difference in total enrollment, the trial durations of the two designs are remarkably close across all eight scenarios, typically differing by less than one month. These results suggest that, under the simulation settings considered, the two designs offer comparable accuracy and efficiency, and the choice between them may be guided primarily by practical considerations discussed in Section 4, such as whether the efficacy assessment window is substantially longer than the DLT window.

Table A3: Comparison of BE-BOIN and BF-BOIN-ET across eight scenarios. Target DLT rate $\phi = 0.25$, five dose levels, $N_{\text{DE}} = 30$, cohort size = 3, DLT window = 3 months, Efficacy window = 3 months, accrual rate = 2 patients/month.

Scenario	Method	MTD sel %	OBD sel %	Patients at MTD	Patients at OBD	Patients overdosed	Total Patients	Trial Duration
1	BE-BOIN	55.3	62.3	11.8	11.3	7.7	30.7	21.6
	BF-BOIN-ET	48.9	60.5	10.8	9.9	9.5	30.2	21.9
2	BE-BOIN	43.8	60.8	8.7	12.3	4.0	33.0	24.1
	BF-BOIN-ET	41.6	61.2	8.7	11.0	5.3	31.4	23.8
3	BE-BOIN	53.4	54.5	9.6	10.7	4.6	37.6	27.7
	BF-BOIN-ET	47.5	53.0	9.2	9.6	5.6	34.8	27.7
4	BE-BOIN	66.8	50.9	8.1	11.0	0.0	38.8	29.1
	BF-BOIN-ET	70.9	54.5	9.1	9.0	0.0	34.2	28.2
5	BE-BOIN	53.9	60.2	11.6	11.6	7.3	28.4	21.8
	BF-BOIN-ET	52.9	62.2	10.9	10.9	8.5	27.2	21.1
6	BE-BOIN	51.7	45.6	9.5	9.5	4.4	36.4	27.7
	BF-BOIN-ET	47.3	47.4	9.3	9.3	5.7	33.5	27.5
7	BE-BOIN	50.9	42.6	10.5	9.3	6.0	37.7	24.7
	BF-BOIN-ET	44.8	43.5	10.0	9.0	8.1	37.5	26.2
8	BE-BOIN	43.9	47.6	8.4	10.4	4.2	38.8	27.2
	BF-BOIN-ET	38.0	48.6	8.0	9.6	5.6	37.5	28.0

MTD sel % denotes the percentage of correct MTD selection. OBD sel % denotes the percentage of correct OBD selection after stage I. Patients overdosed refers to the average number of patients treated above the true MTD. Both designs use the same accrual suspension rules.

References

Barnett H, Boix O, Kontos D, Jaki T. Backfilling cohorts in phase I dose-escalation studies. *Clin Trials*. 2023;20(3):261-268.
